# Supplementary material for: Intrathecal Injection of the Secretome from ALS Motor Neurons Regulated for miR-124 Expression Prevents Disease Outcomes in SOD1-G93A Mice
Source: Biomedicines. 2022 Aug 29;10(9):2120. doi: 10.3390/biomedicines10092120 (PMC9496075; doi:10.3390/biomedicines10092120)
Supplement: Supplementary file 1 [file biomedicines-10-02120-s001.zip › biomedicines-1820264-supplementary.pdf]

Supplementary Tables

Table S1. List of primer sequences used in RT-qPCR.

Gene	Forward primer (5′-3′)	Reverse primer (5′-3′)
**Arginase 1**	CTTGGCTTGCTTCGGAACTC	GGAGAAGGCGTTTGCTTAGTTC
**CX3CL1**	CTCACGAATCCCAGTGGCTT	TTTCTCCTTCGGGTCAGCAC
**CX3CR1**	ATGGGGTCTCTGTCTGCTCT	TACTGGCAATGGGTGGCATT
**Cx43**	ACAGCGGTTGAGTCAGCTTG	GAGAGATGGGGAAGGACTTGT
**Dlg4**	GAGGCTGGCGGCCAGTACACCAG	ACAGAGCAGGCGGTCAG
**Dynein**	GCCTCAGTCTCTGTCCCATC	AAGTCCTGGGGTAAGGTGCT
**GFAP**	CAAACTGGCTGATGTCTACC	GCTTCATCTGCCTCCTGTCTA
**GPR17**	AGCTACGAGGAGTCCACCTG	AGACCGTTCATCTTGTGGCTCT
**IL-10**	ATGCTGCTTGCTCTTACTGA	GCAGCTCTAGGAGCATGTGG
**IL-1β**	CAGGCTCCGAGATGAACAAC	GGTGGAGAGCTTTCAGCTCATA
**iNOS**	ACCCACATCTGGCAGAATGAG	AGCCATGACCTTTCGCATTAG
**Kif5b**	GGTCCTACAGTTGCCACCTA	ATTGAAATACGCCAGGCCCA
**MBP**	CCATCCAAGAAGACCCCACA	CCCCTGTCACCGCTAAAGAA
**MFG-E8**	AGCCTGAATGGTAGGGTTGG	GAGACTGCATCCTGCAACCA
**NeuN**	CCAGGCACTGAGGCCAGCACACAGC	CTCCGTGGGGTCGGAAGGGTGG
**P2RY12**	CACCTCAGCCAATACCACCT	CAGGACGGTGTACAGCAATG
**PLP**	TGGCGACTACAAGACCACCA	GACACACCCGCTCCAAAGAA
**RPL19**	ATGAGTATGCTCAGGCTACAGA	GCATTGGCGATTTCATTGGTC
**S100B**	GAGAGAGGGTGACAAGCACAA	GGCCATAAACTCCTGGAAGTC
**Synaptophysin**	GACGTTGGTAGTGCCTGTGA	GCACAGGAAAGTAGGGGGTC
**TIMP2**	AGCCAAAGCAGTGAGCGAGAAG	GCCGTGTAGATAAACTCGATGTC
**TMEM119**	CCCAGAGCTGGTTCCATAGC	GGGAGTGACACAGAGTAGGC
**TNF-α**	TACTGAACTTCGGGGTGATTGGTCC	CAGCCTTGTCCCTTGAAGAGAACC
**TREM2**	AACTTCAGATCCTCACTGGACC	CCTGGCTGGACTTAAGCTGT

CX3CL1, C-X3-C motif chemokine ligand 1/fractalkine; CX3CR1, c-x3-c chemokine receptor 1; Cx43, connexin 43; Dlg4, discs large MAGUK scaffold protein 4 (encodes for postsynaptic density protein 95, PSD-95); GFAP, *g*lial fibrillary acidic protein; GPR17, G protein-coupled receptor 17; IL-10, interleukin 10; IL-1B, interleukin 1β; iNOS, inducible nitric oxide synthase; Kif5b encodes for the protein kinesin -1 heavy chain; MBP, myelin basic protein; MFG-E8, milk fat globule-epidermal growth factor-factor 8; NeuN, hexaribonucleotide binding protein 3; P2RY12, purinergic receptor p2y12; PLP, myelin proteolipid protein; RPL19, 60S ribosomal L19; RT-qPCR, quantitative real-time reverse transcription polymerase chain reaction; S100B, S100 calcium binding protein B; TIMP2, tissue inhibitor of metalloproteinases 2; TMEM119, transmembrane protein 119; TNF-α, tumor necrosis factor alpha; TREM2, triggering receptor expressed on myeloid cells 2.

Table S2. List of miRNA sequences used in RT-qPCR.

miRNA	Target sequence (5′-3′)
hsa miR-146a-5p	UGAGAACUGAAUUCCAUGGGUU
mmu -miR-155-5p	CTCAGAGAGGTGGAAGACCATGT
hsa miR-21-5p	UAGCUUAUCAGACUGAUGUUGA
hsa-miR-124-3p	UAAGGCACGCGGUGAAUGCC
hsa -miR-125b-5p	UCCCUGAGACCCUAACUUGUGA
SNORD110	Reference gene

miRNA, microRNA; mmu, mouse; hsa, human.

Table S3. List of antibodies used for immunohistochemistry (IHC) or western blot (WB).

	Antibodies	Source	Species	Dilution	
				IHC	WB
Primary	Anti-β-actin	Sigma, A5441	Mouse	-	1:2500
	Anti-GFAP	NovoCastra, GFAP-GA5-6035278	Mouse	1:100	-
	Anti-GFAP	Sigma-Aldrich, G9269	Rabbit	-	1:500
	Anti-Iba1	Wako, 019-19741	Rabbit	1:250	1:500
	Anti-Neu N	Millipore, MAB377	Mouse	1:100	1:100
	Anti-S100B	AbCam, ab52642	Rabbit		1:1000
	Anti-Vimentin	sc-32322, Santa Cruz Biotechnology	Mouse	-	1:200
Secondary	AlexaFluor 488 anti-mouse	Invitrogen, A-10680	Goat	1:500	-
	AlexaFluor 594 anti-rabbit	Invitrogen, A-11012	Goat	1:500	-
	HRP anti-rabbit	Santa Cruz Biotechnology, sc2357	Mouse	-	1:5000
	Mouse-IgGκ BP-HRP	Santa Cruz Biotechnology, sc516102	-	-	1:5000

GFAP, glial fibrillary acidic protein; HRP, horseradish peroxidase; Iba-1, ionized calcium-binding adapter molecule 1; NeuN, hexaribonucleotide binding protein 3; S100B, S100 calcium-binding protein B.

Table S4. Expression of genes, miRNAs and proteins from the muscle and lumbar spinal cord of mSOD1 mice injected with the vehicle or the secretome derived from anti-miR-124-treated mSOD1 MNs (mSOD1 + sec). Results are mean (± SEM) and expressed fold change *vs.* WT + vehicle. One-way ANOVA followed by multiple comparisons Bonferroni post hoc correction was used.

**Markers**	**Fold change** (WT mice + vehicle *vs.* WT mice + vehicle **Mean ± SEM**	**Fold change** (mSOD1 mice + vehicle *vs.* WT mice + vehicle **Mean ± SEM**	***p* values** (*vs.* WT mice + vehicle)	**Fold change** (mSOD1 mice + sec *vs.* WT mice + vehicle) **Mean ± SEM**	***p* values** (*vs.* WT mice + vehicle)	***p* values** (*vs.* mSOD1 mice + vehicle)
**Muscle**						
**Genes**						
NeuN	1.00 ± 0.03	0.45 ± 0.10	0.015	1.18 ± 0.12	0.863	0.003
Synaptophysin	1.06 ± 0.16	0.51 ± 0.10	0.049	1.14 ± 0.14	0.973	0.024
PSD-95	1.01 ± 0.11	0.23 ± 0.08	<0.001	0.78 ± 0.06	0.365	0.011
**Spinal cord**						
**Genes**						
NeuN	1.01 ± 0.08	0.58 ± 0.03	0.004	0.80 ± 0.083	0.181	0.163
Synaptophysin	0.97 ± 0.04	0.95 ± 0.03	>0.999	0.94 ± 0.10	>0.999	>0.999
PSD-95	1.00 ± 0.07	0.51 ± 0.06	0.008	0.70 ± 0.06	0.024	0.211
Dynein	1.00 ± 0.05	0.56 ± 0.07	0.208	0.97 ± 0.17	>0.999	0.166
Kinesin	1.00 ± 0.02	0.72 ± 0.06	>0.999	0.88 ± 0.19	>0.999	0.648
CX3CL1	1.00 ± 0.06	0.76 ± 0.04	0.032	0.73 ± 0.06	0.022	>0.999
MBP	1.00 ± 0.03	0.74 ± 0.08	0.019	1.16 ± 0.04	0.156	<0.001
PLP	1.00 ± 0.03	0.73 ± 0.06	0.006	1.07 ± 0.03	0.927	<0.001
GPR17	1.02 ± 0.13	2.53 ± 0.25	0.008	2.24 ± 0.34	0.038	>0.999
CX3CR1	1.01 ± 0.07	1.53 ± 0.11	0.011	1.50 ± 0.12	0.009	>0.999
Arginase 1	1.01 ± 0.08	0.69 ± 0.04	0.939	1.58 ± 0.32	0.221	0.042
TMEM119	1.04 ± 0.17	2.28 ± 0.28	0.044	2.21 ± 0.35	0.059	>0.999
TIMP2	1.01 ± 0.07	1.49 ± 0.07	0.016	1.51 ± 0.14	0.011	>0.999
P2RY12	1.01 ± 0.07	0.73 ± 0.03	0.028	0.81 ± 0.03	0.121	>0.999
TREM2	1.03 ± 0.12	0.22 ± 0.07	0.004	0.69 ± 0.16	0.291	0.075
MFG-E8	0.97 ± 0.04	0.95 ± 0.03	0.136	0.94 ± 0.10	0.549	>0.999
S100B	1.00 ± 0.05	1.02 ± 0.05	>0.999	1.04 ± 0.06	>0.999	>0.999
GFAP	1.01 ± 0.07	3.00 ± 0.58	0.003	1.49 ± 0.20	0.036	>0.999
CX43	1.00 ± 0.06	1.66 ± 0.11	0.002	1.26 ± 0.13	0.368	0.065
TNF-α	1.00 ± 0.04	2.54 ± 0.54	0.050	2.25 ± 0.38	0.117	>0.999
IL-10	1.01 ± 0.07	0.43 ± 0.08	0.023	1.46 ± 0.18	0.058	<0.001
IL-1β	1.03 ± 0.11	1.16 ± 0.13	>0.999	1.15 ± 0.09	>0.999	>0.999
iNOS	1.02 ± 0.09	0.48 ± 0.09	0.053	1.48 ± 0.19	0.108	<0.001
**Proteins**						
NeuN	1.00 ± 0.12	0.34 ± 0.08	0.051	1.14 ± 0.23	>0.999	0.014
GFAP	1.00 ± 0.05	2.22 ± 0.55	0.263	1.54 ± 0.24	>0.999	0.836
S100B	1.00 ± 0.13	2.75 ± 0.84	0.113	1.50 ± 0.40	>0.999	0.377

Vimentin	1.00 ± 0.14	2.13 ± 0.43	0.046	1.43 ± 0.07	0.845	0.297
Iba-1	1.00 ± 0.08	1.65 ± 0.23	0.030	1.49 ± 0.16	0.132	>0.999
**miRNAs**						
miR-124	1.00 ± 0.05	1.39 ± 0.03	0.003	1.08 ± 0.08	>0.999	0.018
miR-146a	1.01 ± 0.05	1.59 ± 0.28	0.121	1.42 ± 0.18	0.447	>0.999
miR-155	1.00 ± 0.05	2.06 ± 0.52	0.017	0.97 ± 0.11	>0.999	0.017
miR-21	1.00 ± 0.02	1.52 ± 0.20	0.053	1.23 ± 0.15	0.650	0.546
miR-125b	1.00 ± 0.03	1.10 ± 0.06	0.738	1.15 ± 0.07	0.296	>0.999

Results are mean (± SEM) and expressed fold change *vs.* WT + vehicle. One-way ANOVA followed by multiple comparisons Bonferroni post hoc correction was used. CX3CL1, C-X3-C motif chemokine ligand 1/fractalkine; CX3CR1, c-x3-c chemokine receptor 1; Cx43, connexin 43; GFAP, glial fibrillary acidic protein; GPR17, G protein-coupled receptor 17; Iba-1, ionized calcium-binding adaptor molecule 1; IL-10, interleukin 10; IL-1β, interleukin 1β; iNOS, inducible nitric oxide synthase; MBP, myelin basic protein; MFG-E8, milk fat globule-epidermal growth factor-factor 8; miRNA, microRNA; NeuN, hexaribonucleotide binding protein 3; P2RY12, purinergic receptor p2y12; PLP, myelin proteolipid protein; PSD-95, postsynaptic density protein 95; S100B, S100 calcium binding protein B; TIMP2, tissue inhibitor of metalloproteinases 2; TMEM119, transmembrane protein 119; TNF-α, tumour necrosis factor alpha TREM 2, triggering receptor expressed on myeloid cells 2.
